# Clinically relevant molecular hallmarks of PFA ependymomas display intratumoral heterogeneity and correlate with tumor morphology

**DOI:** 10.1007/s00401-023-02682-x

**Published:** 2024-01-24

**Authors:** Swenja Gödicke, Catena Kresbach, Max Ehlert, Denise Obrecht, Lea Altendorf, Karoline Hack, Katja von Hoff, Helena Carén, Viktoria Melcher, Kornelius Kerl, Bernhard Englinger, Mariella Filbin, Kristian W. Pajtler, Johannes Gojo, Torsten Pietsch, Stefan Rutkowski, Ulrich Schüller

**Affiliations:** 1 Department of Pediatric Hematolgoy and Oncology, University Medical Center Hamburg-Eppendorf, Hamburg, Germany; 2 Research Institute Children’s Cancer Center, Hamburg-Eppendorf, Hamburg, Germany; 3 Institute of Neuropathology, University Medical Center Hamburg-Eppendorf, Martinistrasse 52, 20246 Hamburg, Germany; 4 Department of Pediatric Oncology and Hematology, Charité-Universitätsmedizin Berlin, corporate member of Freie Universität Berlin, Humboldt-Universität Zu Berlin, Berlin Institute of Health, Berlin, Germany; 5 Department of Pediatrics and Adolescent Medicine, Aarhus University Hospital, Aarhus, Denmark; 6 Department of Laboratory Medicine, Institute of Biomedicine, Sahlgrenska Center for Cancer Research, Sahlgrenska Academy, University of Gothenburg, Göteborg, Sweden; 7 Department of Pediatric Hematology and Oncology, University Children’s Hospital Münster, 48149 Münster, Germany; 8 Department of Pediatric Oncology, Dana-Farber Boston Children’s Cancer and Blood Disorders Center, Boston, MA 02115 USA; 9 Broad Institute of Harvard and MIT, Cambridge, MA 02142 USA; 10 Department of Urology, Comprehensive Cancer Center, Medical University of Vienna, 1090 Vienna, Austria; 11 Center for Cancer Research and Comprehensive Cancer Center, Medical University Vienna, 1090 Vienna, Austria; 12 Hopp Children’s Cancer Center Heidelberg (KiTZ), 69120 Heidelberg, Germany; 13 Division of Pediatric Neurooncology, German Cancer Research Center (DKFZ), German Cancer Consortium (DKTK), 69120 Heidelberg, Germany; 14 Department of Pediatric Oncology, Hematology and Immunology, Heidelberg University Hospital, 69120 Heidelberg, Germany; 15 Department of Pediatrics and Adolescent Medicine, Comprehensive Center for Pediatrics and Comprehensive Cancer Center, Medical University of Vienna, 1090 Vienna, Austria; 16 Institute of Neuropathology, DGNN Brain Tumor Reference Center, University of Bonn Medical Center, Bonn, Germany

**Keywords:** Ependymoma, Intratumoral heterogeneity, 1q gain, 6q loss, Morphology, DNA methylation

## Abstract

Posterior fossa type A (PF-EPN-A, PFA) ependymoma are aggressive tumors that mainly affect children and have a poor prognosis. Histopathology shows significant intratumoral heterogeneity, ranging from loose tissue to often sharply demarcated, extremely cell-dense tumor areas. To determine molecular differences in morphologically different areas and to understand their clinical significance, we analyzed 113 PF-EPN-A samples, including 40 corresponding relapse samples. Cell-dense areas ranged from 0 to 100% of the tumor area and displayed a higher proportion of proliferating tumor cells (*p* < 0.01). Clinically, cell density was associated with poor progression-free and overall survival (*p*_PFS_ = 0.0026, *p*_OS_ < 0.01). Molecularly, tumor areas with low and high cell density showed diverging DNA methylation profiles regarding their similarity to distinct previously discovered PF-EPN-A subtypes in 9/21 cases. Prognostically relevant chromosomal changes at 1q and 6q showed spatial heterogeneity within single tumors and were significantly enriched in cell-dense tumor areas as shown by single-cell RNA (scRNA)-sequencing as well as copy number profiling and fluorescence in situ hybridization (FISH) analyses of different tumor areas. Finally, spatial transcriptomics revealed cell-dense areas of different tumors to be more similar than various different areas of the same tumor. High-density areas distinctly overexpressed genes encoding histone proteins, WNT5A, TGFB1, or IGF2. Relapsing tumors displayed a higher proportion of cell-dense areas (*p* = 0.036), a change in PF-EPN-A methylation subtypes (13/32 patients), and novel chromosome 1q gains and 6q losses (12/32 cases) compared to corresponding primary tumors. Our data suggest that PF-EPN-A ependymomas habor a previously unrecognized intratumoral heterogeneity with clinical implications, which has to be accounted for when selecting diagnostic material, inter alia, by histological evaluation of the proportion of cell-dense areas.

**Supplementary Information:**

The online version contains supplementary material available at 10.1007/s00401-023-02682-x.

## Introduction

Ependymomas are well-circumscribed tumors of the central nervous system (CNS) that originate from ependymal cells. Histopathology reveals glial neoplasms that can arise in the supratentorium, the posterior fossa, and along the spinal cord [25]. Ependymomas of the different locations are further divided into ten molecular types with distinct molecular and clinical characteristics, based on their DNA methylation profiles [25, 35, 36]. Ependymomas can occur at all ages but predominantly arise in pediatric patients where they constitute the third most common primary brain tumor. In pediatric patients, 90% of ependymomas are located intracranially, with two-thirds occurring in the posterior fossa. The posterior fossa ependymomas of the molecular subgroup A (PF-EPN-A) mainly affect infants and young children and have a dismal prognosis [36]. PF-EPN-A requires intensive multimodal therapy comprising maximum safe surgical resection, postoperative radiotherapy and in some cases chemotherapy. The aggressiveness of this regime, while improving survival, is associated with severe treatment-related morbidity including reduced neurocognitive outcomes [47]. For improved overall outcomes and quality of life, better risk stratification of PF-EPN-A patients and innovative additional therapy options are urgently needed. Up to date, the strongest clinical prognostic factor remains the extent of resection [41]. While the genome of PF-EPN-A is generally considered relatively stable, approximately 19% of tumors show chromosome 1q gain and 9% 6q loss as the most frequent copy number variations (CNV) [36]. Both CNV were shown to be strong independent negative prognostic factors. Moreover, patients, whose tumors harbor combined chromosome 1q gain and 6q loss have a significantly worse prognosis than those with chromosome 1q gain or 6q loss only [3, 20]. RNA-, whole genome, and whole exome sequencing of PF-EPN-A revealed no frequent fusion transcripts or somatic single-nucleotide variants (SNV) and a low general mutation rate with only a few tumors harboring mutations in genes encoding H3, ACVR1, or EZHIP [37, 39]. Thus, PF-EPN-A are generally assumed to be epigenetically driven. PF-EPN-A tumor cells are characterized by a global loss of the repressive histone 3 lysine 27 trimethylation (H3K27me3) that is not found in the prognostically more favorable PF-EPN-Bs. An aberrant expression of the enhancer of EZHIP or, mutually exclusive, rare H3K27M mutations were identified as the cause for this loss of H3K27me3 in PF-EPN-A cases. Both mechanisms inhibit EZH2, the functional enzymatic component of polycomb repressive complex 2 (PRC2) that normally catalyzes H3K27 trimethylation. Since in the CNS and its tumors EZHIP expression is almost exclusive to PF-EPN-A, it is considered as a reliable immunohistochemical (IHC) marker. These markers may also constitute a possible therapeutic target, as the respective mechanisms are considered to be drivers of PF-EPN-A tumorigenesis [2, 4, 15, 16, 34, 35, 42].

PFA ependymomas have been described to form two robust methylation subgroups, PF EPN-A1 and 2. More recently, a further subdivision in nine subtypes 1a-f, 2a-c was proposed, describing them as distinct in DNA-methylation, clinical characteristics, WHO-grading, and prognosis [35]. However, in the present study we show that heterogeneity is not restricted to intertumoral differences in methylation. Histopathologically, PF-EPN-A show a striking range of cell density, not only between different patients and tumors but also within one tumor, often as sharply demarcated areas of higher and lower cell density. We combined histomorphological, immunohistochemical, and molecular approaches to objectify and further characterize this heterogeneity concerning cell density and revealed possible clinical implications.

## Material and methods

### Samples and clinical data

**Fig. 1:**
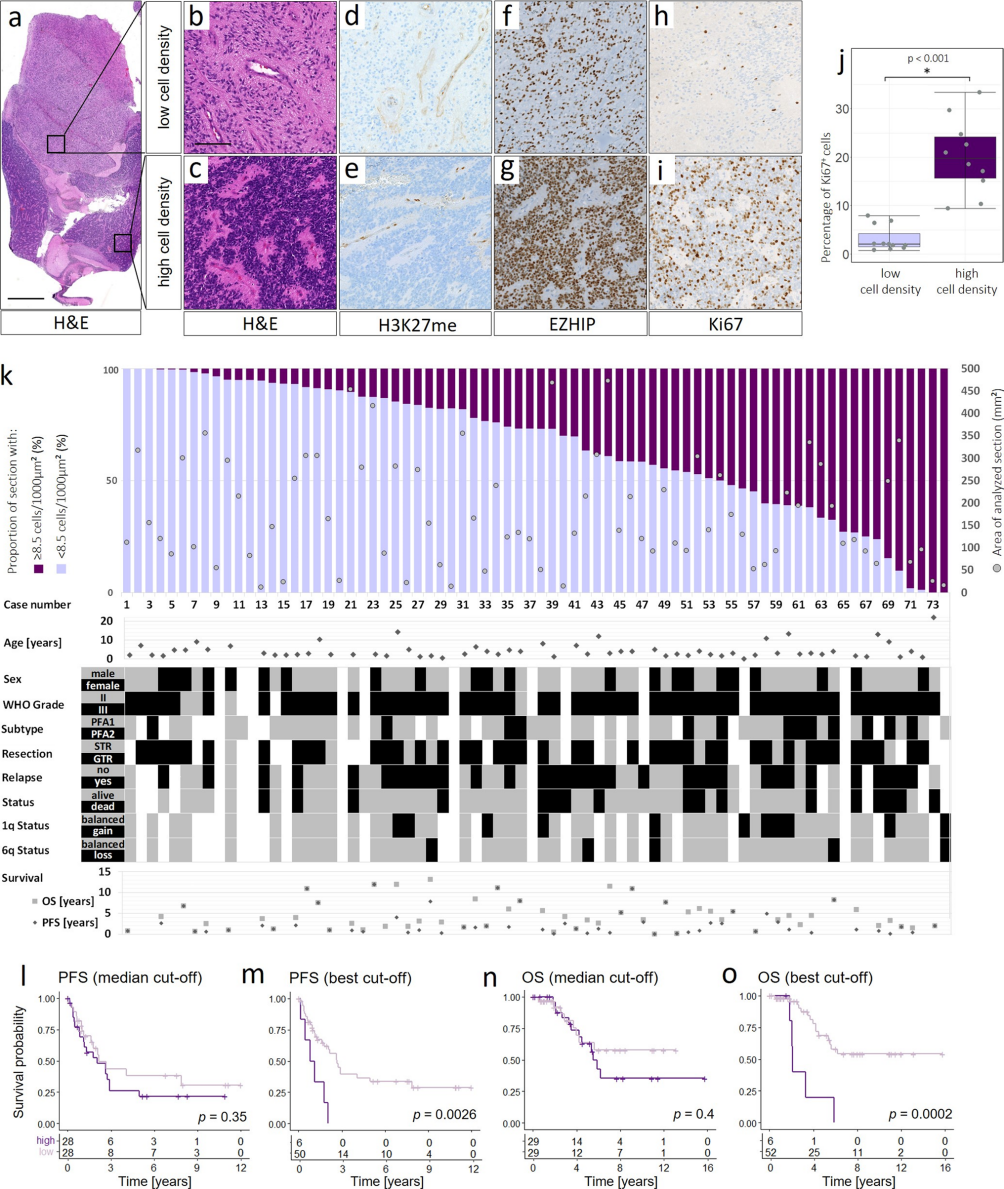
Histological and clinical characterization of primary PF-EPN-A tumors harboring varying extents of high- and low-cellularity areas. **a**–**c** H&E staining of one PF-EPN-A tumor with areas with low (**b**) and high (**c**) cell density. **d**–**i** H3K28me, EZHIP, and Ki67 staining of the corresponding areas. **j** Proportion of Ki67-positive cells in cell-dense and less cell-dense areas in 11 PFA ependymomas. In all tumors the entirety of all areas with low and high cell density within the tumor was analyzed for one data point, respectively. **k** Percentage of areas with low and high cell density in 74 PFA ependymomas, clinical data, and methylation subgroups. (Scale bars correspond to 1.5 mm in a and to 125 µm in **b**–**i**). **l**–**o** Kaplan–Meier-Curves of the progression-free and overall survival in 58 tumors (all tumors of the cohort in (**k**) with available clinical data). **l**, **n** Comparison of the 29 tumors with a lower proportion of cell-dense areas to the 29 tumors with a higher proportion of cell-dense areas, groups classified as below and above the median and **m**, **o** comparison of the six tumors with the highest proportion of cell-dense areas (#66, #68, #69, #70, #71, and #73) to the rest of the cohort. Here, group allocation represents the cut-off with the highest resulting significance for both analyses and equals the comparison between all tumors with ≥ 70% to those with < 70% cell-dense areas

Inclusion criterion for this study was a formal diagnosis as an ependymoma type PF-EPN-A attained in routine diagnostics, as well as available FFPE tumor tissue. 74 PF-EPN-A cases were included in our initial cohort. For 58/74 cases clinical data were available, 51 of these were included in different clinical trials (HIT2000 *n* = 30, SIOP-Ependymoma II Version 3.0 *n* = 20, SKK92 *n* = 1) and seven were additional patients of the University Hospital Hamburg-Eppendorf. Detailed clinical and patient characteristics are shown in Fig. 1 and in Suppl. Table 1. As validation cohort, H&E slides and survival data of a total of 68 additional PF-EPN-A cases from the HIT2000 trial were analysed. The respective data are listed in Suppl. Table 2, online resource. The use of clinical data and tumor material for research was always in accordance with ethical standards and regulations at the University Medical Center Hamburg-Eppendorf. The respective clinical trials were approved by the responsible ethics committee. For trial patients, informed consent was obtained in accordance with the Declaration of Helsinki.

### Histomorphological and immunohistochemical evaluation

Paraffin sections of 2 µm were stained in hematoxylin and eosin (H&E) according to standard protocols. Stained slides were scanned on a NanoZoomer Digital Slide Scanner (Hamamatsu, Japan). Scanned slides were analyzed digitally after defining the total area and proportion of cell-dense and less cell-dense areas using QuPath digital image analysis (version 0.2.3). For the QuPath protocol, after the normalization of the stain vectors across all samples, a threshold that distinguished areas with more and less than 8.5 cells per 1,000 µm^2^ with a resolution of 28.88 µm/px, was created. This cut-off used for all samples was chosen based on the evaluation of ten randomly chosen cases and constitutes the cut-off that best depicted the visually determined sharp demarcation lines in these samples. We analysed one slide per tumor. In tumors with more material available, we examined multiple slides, at least one per available FFPE block of the respective tumor.

Immunohistochemical staining of paraffin sections was performed on an automated Ventana staining instrument using the following antibodies: H3K27me3 (cell signaling, 9733, 1:50), EZHIP (Sigma-Aldrich, #HPA004003, 1:50), and Ki67 (Abcam, ab15580, 1:100). In eleven cases, the percentage of Ki67-positive cells of all cells in the cell-dense and less cell-dense areas was determined using the “positive cell detection” feature in QuPath digital image analysis. One of the eleven tumors analysed consisted of low cell density areas only. Undetected positive cells were added to the calculation manually.

### Fluorescent in situ hybridization (FISH)

Dual colour interphase FISH was performed on FFPE sections using commercial probe sets. For the evaluation of a potential chromosome 1q gain, a spectrum green labeled probe was used for test locus 1q25.3 and a spectrum orange labelled probe for control locus 1p36.31 (ZytoLight). For the evaluation of a potential chromosome 6q loss, a spectrum green labelled probe was used for test locus *ESR1* at 6q25.1 and a spectrum orange labelled probe for the alpha satellite centromeric region of chromosome 6 (D6Z1) (ZytoLight). Signals were scored under a fluorescence microscope in 100 non-overlapping, intact nuclei under oil-immersion. If a cell showed three or more signals of the 1q test probe or the ratio of test probe-control probe inside one nucleus was more than one, signals were scored as chromosome 1q gain. If a cell showed one or less signals of the 6q test probe and/or the ratio of test probe-control probe inside one nucleus was ≤ 0.5, signals were scored as chromosome 6q loss. Tumor signals were scored as gains or losses for the whole area when at least 10% of the cells showed the gain or loss, as previously described [22, 28].

### Survival analysis

All statistical analyses and data visualizations were performed using the R programming language. (R version 4.2.2). Survival analysis was performed using the survminer package (version 0.4.9). Overall survival (OS) and progression-free survival (PFS) curves were plotted using the Kaplan–Meier method. PFS was measured from the date of surgery to the date of relapse. OS was calculated from the date of surgery to the date of death. Patients without documented progression for PFS and without confirmed death for OS were censored at their last follow-up visit. The *p*-value was calculated using the log-rank test, *p* < 0.05 was considered significant. Groups for survival analysis were split at the median for the first, and best cut-off for the second analysis. Best cut-off was defined as the group allocation that includes all cases and results in the highest significance (the smallest *p*-value). It was determined using a brute-force search approach/exhaustive algorithm in R.

### DNA methylation profiling

DNA methylation profiling of in-house samples was performed using “MethylationEPIC” BeadChip arrays (Illumina). Reference cohort methylation data were derived from the CNS tumor reference cohort (514 ependymoma cases, 240 of which were PF-EPN-A cases) established by Capper et al. in 2018 (GEO accession GSE109381) [7]. In addition, we integrated data of the subtype SP-EPN-MYCN from Ghasemi et al. 2019 (6 cases, EPIC) and Raffeld et al. 2020 (9 cases, 450 K) [11, 40]. Concerning the pairs of primary and first relapse tumors evaluated, we included patients of the University Hospital Hamburg-Eppendorf, cases from the Capper et al. cohort [7], and two primary relapse pairs (R13 and R14) originally published by Wenger et al. in 2022 (GEO accession GSE247880) [46]. Detailed information on the analysed primary relapse pairs is presented in Suppl. Table 3. Type and subtype classification and copy number profile analysis were undertaken on the Heidelberger Brain Tumor Classifier, version 12.5, available via www.molecularneuropathology.org [7]. For methylation-derived copy number variations (CNV), the individual copy number profiles were manually examined. The cut-off for a chromosome 1q gain and/or 6q loss was 0.25 and − 0.25, respectively.

Uniform manifold approximation and projection (UMAP), *t*-distributed stochastic neighbor embedding (*t*-SNE), and unsupervised heatmap transformation and plotting were performed using the R packages minfi (version 1.44.0), umap (version 0.2.9.0), ggplot2 (version 3.4.0) and ComplexHeatmap (version 2.14.0). The 10,000 most variably methylated CpG sites were selected by standard deviation. Probes on sex chromosomes, probes with a detection *p*-value of or above 0.01, probes with SNPs at the CpG site, cross-reactive probes, and probes, that were not represented in both the EPIC and the 450 k array were excluded from the analysis. Adjustment for batch effects between array types was performed. *T*-SNE plots were generated using Rtsne with max_iter = 100,000, theta = 0, perplexity = 20. Unsupervised hierarchical clustering was performed with Pearson distancing and average linkage. The amount of tumor-infiltrating lymphocytes was estimated using the DIMEimmune method [43].

### Single-cell RNA sequencing (ScRNASeq)

ScRNASeq data were obtained from Gene Expression Omnibus (GEO) accession GSE141460 [13]. The count matrix provided was processed according to the standardized Seurat workflow [14] using the R package Seurat (version 4.3.0). Cell barcodes were filtered out if they had less than 200 and more than 2500 genes as well as cells with more than 5% of UMIs derived from mitochondrial genes. After normalization und dimensional reduction via Principal Component Analysis (PCA) and UMAP, we used Harmony alignment using the R package harmony (version 0.1.1) [23] to further integrate the given data. For visualization, we chose *t*-SNE. Subsequently, cell types were assigned depending on differential expression and marker genes per cluster. CNV were projected by mapping gene expression alterations on a single cell level via the R package inferCNV (version 3.17). Results were then added back to the Seurat object to visualize potential chromosome gains and losses in *t*-SNE plots as well as violin plots.

### Spatial transcriptomics

For Nanostring GeoMx Digital Spatial Profiling (DSP), areas with both high and low cell density areas were selected on H&E-stained slides from FFPE tissues. Tissue Sects. (5 µm) of the corresponding areas were processed according to published protocols. [29] RNA-preserving antigen retrieval was performed, followed by RNA target exposure with proteinase K. In situ hybridization of all targets (*n* = 18,677) on the tissue sections was performed overnight at 37 °C, followed by stringent washes to remove off-target probes. Subsequently, antibodies against GFAP (Novus, NBP2-33184DL594, 5 µg/ml), Ki-67 (CST, 9027S, 6 µg/ml), and OLIG2 (Millipore, AB9610, 1:200) were used to visualize target areas as well as DAPI for nuclei counterstains. Three areas of low and high cell density each were selected with the custom polygon region of interest (ROI) tool for each case. These selected areas were illuminated individually via UV light on a GeoMx DSP, resulting in photocleavage of oligonucleotides present within each ROI. Each aspirate contained photocleaved DNA oligos comprising an analyte identifier, a unique molecular identifier (UMI) barcode, and a primer binding site. Illumina adapter sequences and unique dual-sample indices were added when PCR was performed on the aspirates. Sequencing was performed using Illumina's NovaSeq 6000 with the S4 Reagent Kit v1.5 35 cycles flowcell, and paired-end reads. Signals were then scaled to nuclei count using the geometric mean method for each ROI and normalized for each probe against the 75th percentile of the cumulative signal of the respective ROI. Background correction was performed by geometric mean method against the average background calculated by signal-to-noise ratio using the negative probe counts. For #28 low, #41 high, #54 high and #59 high each, one out of the three samples had to be excluded after running quality control (QC) due to a too low percentage of stitched and aligned reads and/or a too low negative probe count. For subsequent transcriptome analysis, gene expression in all remaining low cell density probes was compared to all remaining high cell density probes using the DESeq2 package (version 1.38.1) within R. Unsupervised clustering was performed using the ConsensusClusterPlus package (version 1.62.0) and ComplexHeatmap (version 2.14.0).

## Results

### PF-EPN-A harbor a great histological heterogeneity with a higher proportion of cell-dense areas correlating with poorer outcomes

PFA ependymomas show a pronounced histomorphological variability and heterogeneity, inter- as well as intratumorally. Tumor cell density may vary within a tumor and sometimes shows well-demarcated nodules of densely packed tumor cells surrounded by loose glial tissue composed of diffusely distributed tumor cells (Fig. 1a–c). To ensure that the cells in high and low cell density areas are indeed PF-EPN-A tumor cells, we evaluated H3K27me3- and EZHIP-stained sections (Fig. 1d–g). The great majority of cells in the analyzed sections were H3K27me3-negative and EZHIP-positive in both cell-dense and less cell-dense areas, the only exceptions being endothelial cells and occasional immune cells. Ki67 staining showed a significantly higher percentage of proliferating tumor cells in the cell-dense areas (*n* = 10) compared the lower cell-density areas (*n* = 11, Fig. 1h–j, p < 0.001). We next aimed to quantify the observation of variable cell-density and distinct cell-density areas within PF-EPN-A tumors using a digital threshold. The proportion of cell-dense (> 8.5 cells/1000 µm^2^) areas of the total analyzed tumor area was highly variable on a continuum ranging from 0 to 100% of the analyzed sections (*n* = 83, Fig. 1k). Next, we investigated possible correlations between histomorphology and clinical characteristics of the tumors. The clinical characteristics of our cohort are summarized in Suppl. Table 1. There was no obvious correlation of either sex, WHO grade at diagnosis, PFA1/2 subtype affiliation, extent of resection, relapse, death, or chromosome 1q gain or 6q loss with the proportion of cell-dense areas. Separating our cohort into two groups at the median cut-off for cell density showed no significant difference in either PFS or OS (*p*_PFS_ = 0.35; *p*_OS_ = 0.4, Fig. 1l, n). However, separating the groups at the best cut-off, resulting in a comparison of six predominantly high-cellularity cases vs. 50 (PFS) or 52 (OS) predominantly low-cellularity cases, indicated significantly worse PFS (*p*_PFS_ = 0.0026) and OS (*p*_OS_ < 0.001) of predominantly cell-dense cases (Fig. 1m, o). We deemed reviewing and verifying the results in an independent validation cohort prudent and established a second cohort, consisting of 68 gross totally resected PF-EPN-A for which we repeated the analyses. Survival data and proportions of cell-dense areas of this validation cohort are summarized in Suppl. Table 2, online resource. Survival analysis confirmed a significantly worse overall and progression-free survival for cases with a high proportion of cell-dense areas, both when separating the groups at the median (*p*_PFS_ = 0.00021; *p*_OS_ = 0.00085), and the best cut-off (*p*_PFS_ < 0.0001; *p*_OS_ = 0.00085) (Suppl. Figure 1, online resource).

### High and low cell density areas show differences in DNA methylation and may be assigned to different molecular subtypes

**Fig. 2:**
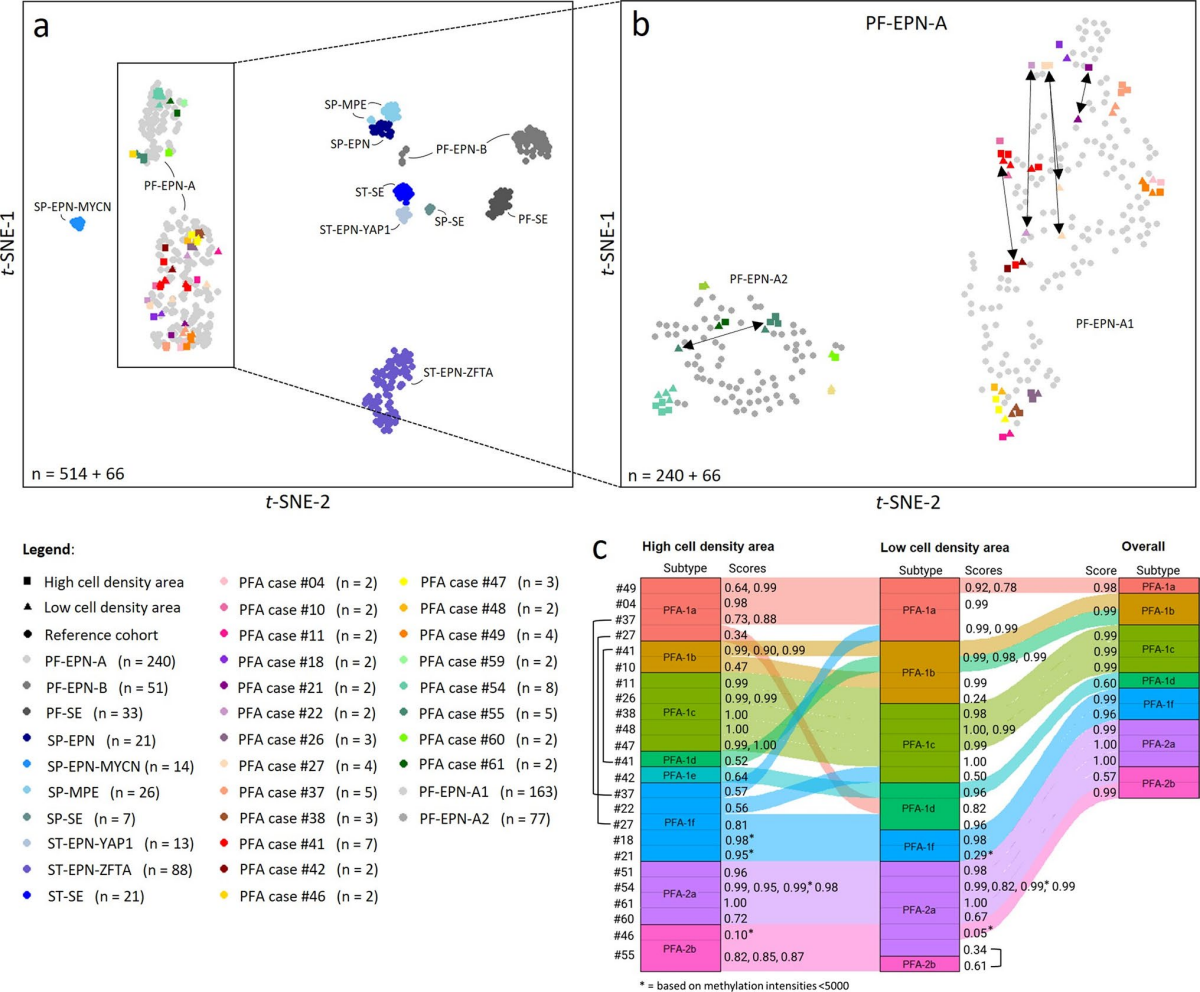
Methylation subtype changes depending on the localization of sample collection within the 15 examined PFA-cases. **a** Unsupervised *t*-SNE analysis of low and high cell density areas of 21 heterogeneous PFA ependymomas together with a reference cohort consisting of 515 samples compromising 10 distinct molecular ependymoma and subependymoma entities. **b** Separate *t*-SNE analysis of the same heterogeneous PFA ependymoma samples and PFA ependymomas of the reference cohort as in **a**. **c** Subtype changes depending on where in the 15 examined PFA-cases the sample for the methylation analysis was taken. Scores according to v12.5 of the DKFZ brain tumor methylation classifier (www.molecularneuropathology.org). Scores listed equal the best match. Multiple scores for one case each equal the score of one of multiple independently taken samples from the same area from different parts of the same tumor. Brackets ([) indicate samples from the same tumor and area that scored highest for different subtypes. Marked scores (*) are based on methylation intensities below 5,000

Next, we assessed possible differences in global DNA methylation between morphologically different tumor areas in 21 cases of our initial cohort. Methylation data of cell-dense and less cell-dense samples of 21 PF-EPN-A tumors (66 samples) were plotted together with a reference cohort of 514 ependymomas and, in a separate second analysis, with 240 PF-EPN-A using *t*-SNE (Fig. 2a, b), UMAP, and unsupervised hierarchical clustering analysis (Suppl. Figure 2a, b, online resource). Overall, the methylation signature of the low and high cell density areas within one tumor was similar, but not identical. For some tumors, the samples plotted clearly apart, suggesting distinct DNA methylation profiles in low and high cell density areas within PF-EPN-A ependymomas (see double-headed arrows in Fig. 2b). We then investigated for potential methylation subtype changes from low to high cell density areas of the same tumors according to the subtypes defined by Pajtler et al. [35]. In 9/21 cases, cell-dense and less cell-dense areas scored best for different subtypes in at least one sample according to the DKFZ brain tumor methylation classifier (Fig. 2c). Subtype classification of FFPE tumor material obtained for routine diagnostics without paying regards to cellularity (available for *n* = 13 out of the 21 cases), coalesced that of the lower cell density area in all but two cases (#46, #60). Taken together, we found that high and low-density areas in morphologically heterogeneous PF-EPN-A both reliably receive the highest scores for PF-EPN-A but might differ in the assigned PF-EPN-A subtype.

The tumor microenvironment, including tumor-infiltrating lymphocytes (TIL) contributes significantly to genesis and progression of CNS tumors [5, 27]. Additionally, differences in the amount of immune infiltration might possibly alter the DNA methylation profiles and account for ambiguous classification results. As an approach to evaluate immune infiltration and search for possible differences between different cell density areas, we used the DIMEimmune (Differential Methylation Analysis for Immune Cell Estimation) method implemented by Safaei et al. [43]. We estimated and compared the CD4 + and CD8 + T cell abundance as well as tumor-infiltrating lymphocytes (TILs) scores in high and low cell density areas of the 21 heterogenous PF-EPN-A with available DNA methylation data. There was no significant difference in immune infiltration between high and low cell density areas in our analysis (DIME-TIL (*p* = 0.56), DIME-CD4 + (*p* = 0.63) and DIMECD8 + score (*p* = 0.81)) (Suppl. Figure 3, online resource).

### Prognostically relevant CNV show a high intratumoral heterogeneity with enrichment in high cell density areas

**Fig. 3:**
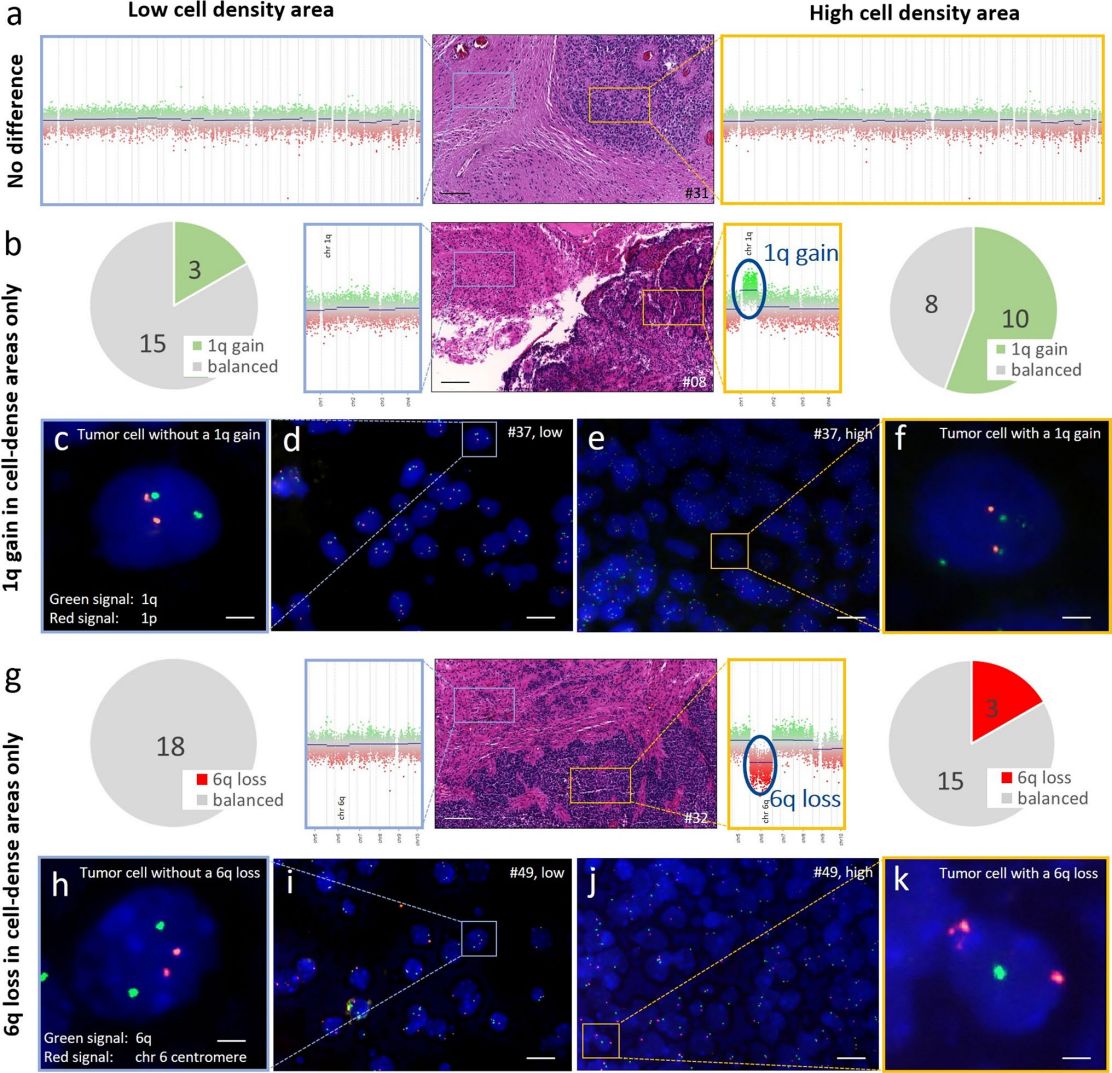
Prognostically relevant copy number variations are enriched in high cell density areas. **a**, **b**, **g** CNV profiles derived from microdissected tumor areas with low and high cell density in 3 heterogenous PFA ependymomas. Pie charts represent how many of the cases showed the indicated CNV in the respective area (*n* = 18). Scale bars correspond to 100 µm. **c**–**f, h**–**k** Fluorescence in-situ hybridization analysis on the (**c**–**f**) 1q status (green) and (**h**–**k**) 6q status (green) in the less cell-dense (**c**, **d**, **h**, **i**) and cell-dense (**e**, **f**, **j**, **k**) area of one tumor each. Scale bars correspond to approximately 2 µm in **c**, f, **h**, and k and to 10 µm in **d**, **e**, **i**, and **j**

Next, we investigated possible differences in the chromosome 1q and 6q status of cell-dense and less cell-dense areas. First, we analyzed copy number variation profiles inferred from DNA methylation data for 18 cases from our initial cohort. In the low cell density areas, 3/18 cases had a 1q gain and 0/18 cases had a 6q loss. In the higher cell density area, 10/18 displayed a chromosome 1q gain and 3/18 a 6q loss. Two-tailed Fisher’s exact test yielded a *p* = 0.035 for chromosome 1q gain and a *p* = 0.2286 for chromosome 6q loss. In total, in 8/18 cases, a 1q gain (*n* = 6) and/or a 6q loss (*n* = 3) was found in the cell-dense areas that were not detected in the lower cell density areas of the same tumor (Fig. 3a, b, g). FISH analysis confirmed these findings in 20 (1q) and 10 (6q) exemplarily analyzed cases, 13 (1q) and 8 (6q) of which were not already part of the performed DNA methylation analysis (Fig. 3c-f, h–k).

**Fig. 4:**
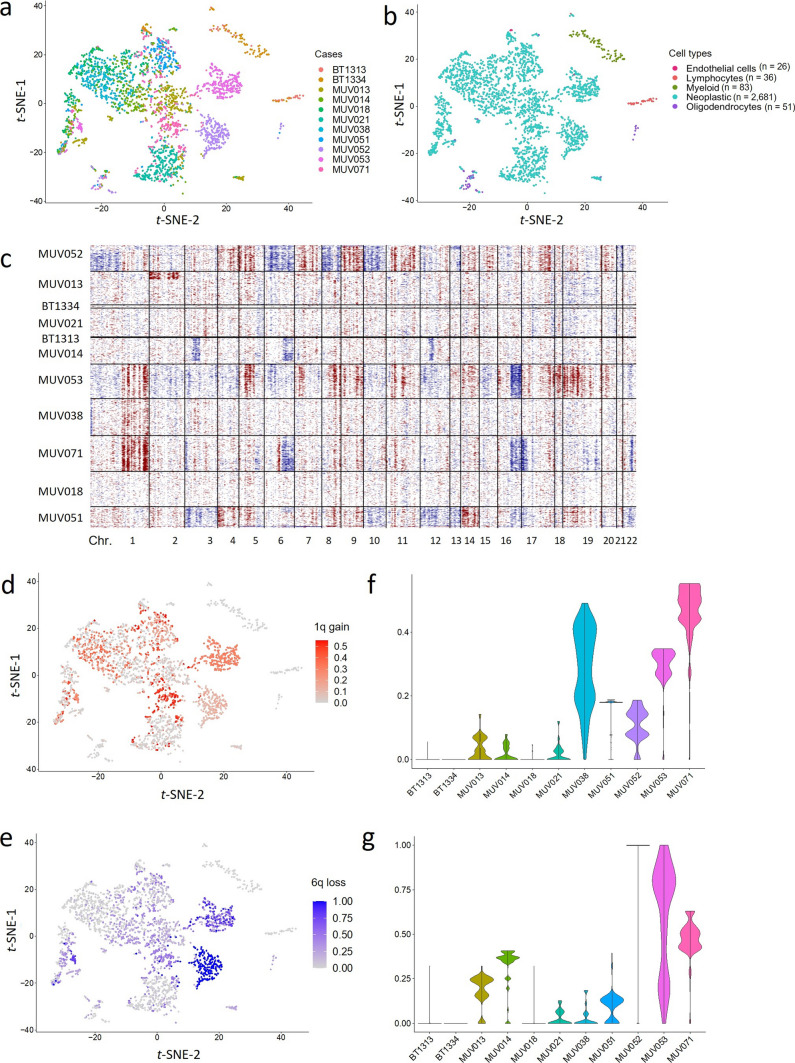
ScRNAseq analysis of 12 PFA ependymoma samples reveals inter- and intratumoral heterogeneity regarding CNV. **a** Clustering of 12 PFA samples (*n* = 3,069 cells). **b** Visualization of different biological cell populations in 12 PFA samples. **c** Inferred copy number alterations in 12 PFA samples. **d** Visualization of 1q gain levels in 12 PFA samples clustered according to their global gene expression. **e** Visualization of 6q loss levels in 12 PFA samples clustered according to their global gene expression. **f** Sample-wise comparison of 1q gain levels in PFA tumor cells. **g** Sample-wise comparison of 6q loss levels in PFA tumor cells

We re-analyzed publicly available scRNA Seq data of eleven PF-EPN-A cases to further investigate the expression profiles of possible tumor subclones and the distribution of copy number changes. In a *t-*SNE analysis, the cells of each tumor were predominantly plotted together (Fig. 4a), indicating a patient-specific gene expression pattern. The samples included only very few non-neoplastic cells that could influence and potentially distort the results (Fig. 4b). CNV inferred from gene expression alterations on a single cell level indicated 1q gain in 3/11 cases and 6q loss in 3/11 cases. (Fig. 4c). Both, chromosome 1q gain and 6q loss were distributed very heterogeneously not only between different PF-EPN-A tumors but also intratumorally (Fig. 4d, e). Sample-wise plotting of the chromosome 1q gain and 6q loss levels more specifically revealed the intratumoral heterogeneity (Fig. 4f, g). For both chromosome 1q gain and 6q loss, there were tumors displaying a relatively similar level of the respective CNV in all tumor cells (e.g., MUV021, BT1334). In others, the level of the CNV ranged from 0 to 1.00, with varying distribution patterns (sometimes evenly distributed, like in MUV038 for chromosome 1q gain and MUV053 for 6q loss, sometimes accentuated on one level, like in MUV071).

### High and low cell density areas in PF-EPN-A have a distinct transcriptome

**Fig. 5:**
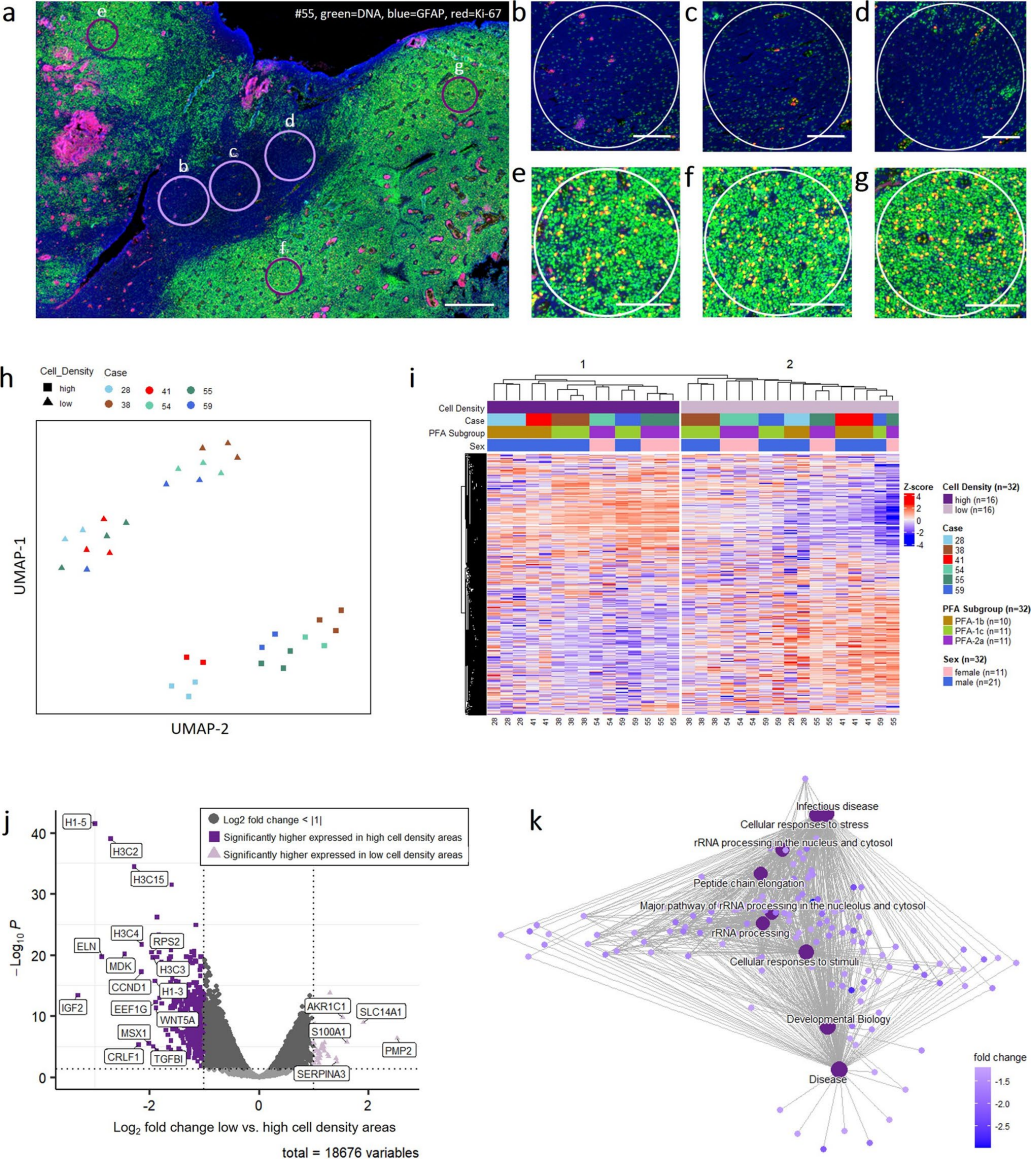
Spatial Transcriptomics reveal distinct transcriptomes of high and low cell density areas. **a**–**g** Fluorescence histology images of one out of six cases (#55) for which spatial transcriptomics were performed. Overview (**a**) and the analyzed regions of interest (ROIs) of low (**b**–**d**) and high (**e**–**g**) cell density, three each. Scale bars correspond to approximately 1 mm in a and to 250 µm in **b**–**g**. **h** Unsupervised UMAP analysis of gene expression in low and high cell density areas of 6 heterogeneous PFA ependymoma (2–3 samples per case and cell density). **i** Unsupervised heatmap of the same samples as in **a** considering the whole gene set. **j** Volcano Plot of differential gene expression between high and low cell density areas of the same 6 heterogenous PFA ependymoma as in **a**, **b**. **k** Network Plot of the top 9 enriched pathways in a GSEA low vs. high cell-density areas of the same samples as in **a**–**c**, pathways shown are enriched in the cell-dense areas

Since both, DNA methylation and CNV, greatly influence gene expression, our next step was to investigate possible differences in the transcriptome of high and low cell density areas. We performed spatial transcriptomics of six PF-EPN-A tumors, analyzing three ROIs per cell density category in each tumor (exemplary fluorescence scan with the respective ROIs in Fig. 5a–g). Both UMAP visualization and unsupervised hierarchical clustering of the whole gene set confirmed that high and low cell density samples formed distinct transcriptional groups (Fig. 5h, i). All samples clustered according to cell density irrespective of case identity, PF-EPN-A subtype, or sex of the patient (Fig. 5i). Differential gene expression analysis revealed distinct gene expression in high compared to low cell density areas (Fig. 5j). Upregulated genes in the high cell density areas compared to low cell density areas most prominently included a large number of members of the histone and ribosomal protein families. Further genes significantly upregulated in the high cell density areas have previously been described as being associated with tumorigenesis, -progression, resistance to chemotherapy, and radiation sensitivity in malignancies, including CNS tumor entities like ependymomas and glioblastomas. Some of the most noteworthy were IGF2 [21, 31], MDK [10, 18, 19, 32], TBFBI [8, 38], WNT5A [6], LDHA [21, 30, 45] and CCND1 [24]. Gene set enrichment analysis (GSEA) revealed a multitude of upregulated pathways in the cell-dense areas (Fig. 5k), suggesting that high cell density areas in PF-EPN-A are generally more transcriptionally active and might play a more active role in tumor propagation.

### Primary tumors and their relapses differ in respect to cell density, DNA methylation and frequency of chromosome 1q gains and 6q losses

**Fig. 6:**
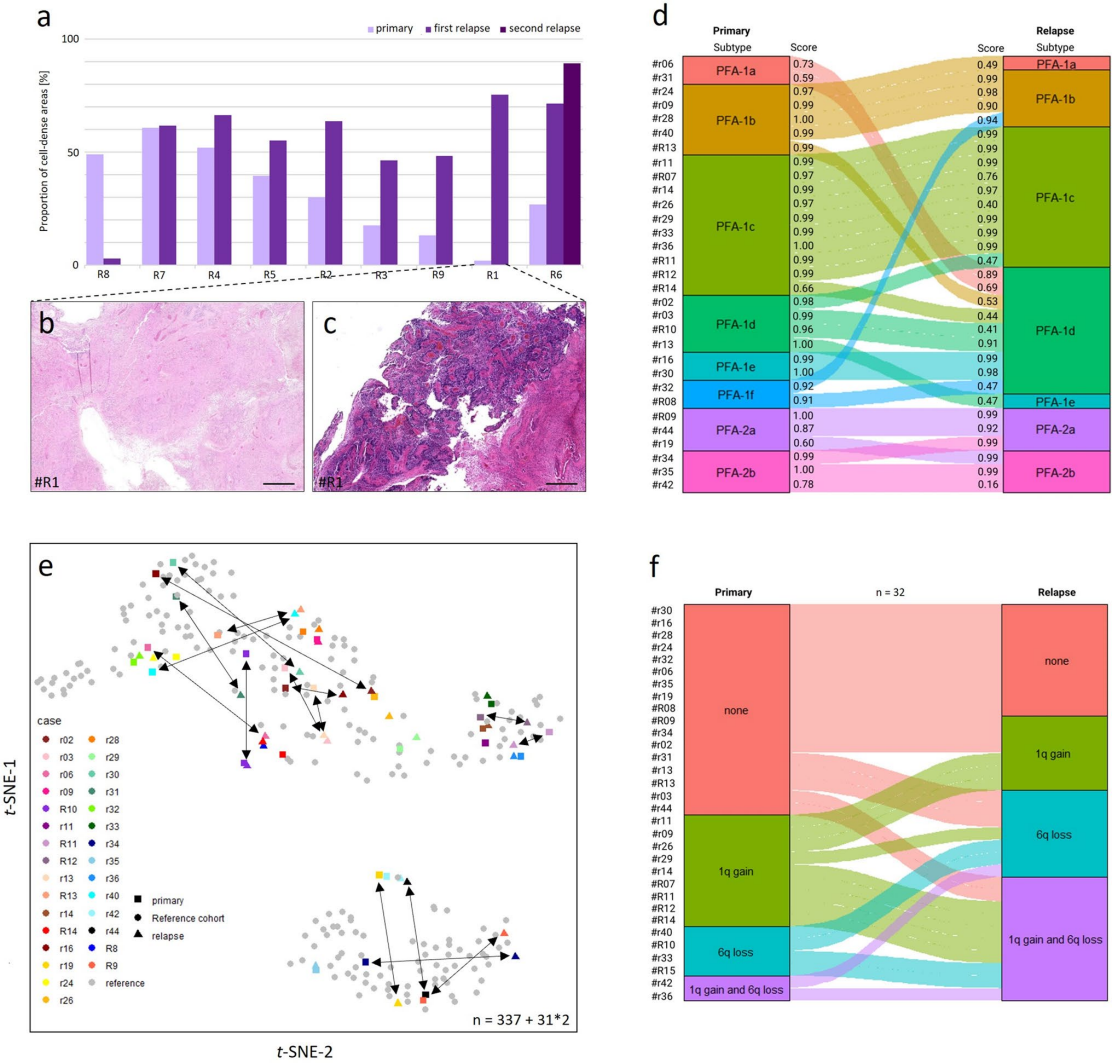
Cell density, DNA methylation and CNVs change at recurrence. **a** Progression of cell density in 9 PFA-ependymoma relapses. **b**, **c** Representative picture sections from H&E stainings of a primary tumor (**b**) and its relapse (**c**). Scale bars correspond to 1.5 mm in **b** and **c**. **d** Subtype changes in 31 PFA-ependymoma relapses in comparison to the primary tumors. Scores according to V12.5 of the DKFZ brain tumor methylation classifier (www.molecularneuropathology.org). **e** Unsupervised *t*-SNE analysis of 31 primary tumors and their relapses together with a reference cohort consisting of 337 PFA samples. **f** 1q gain and 6q loss in 32 primary tumors and their relapses. Alluvial plot shows the change in 1q and 6q status taken together per case from the primary tumor to the first relapse

CNS tumor relapses tend to acquire more aggressive biology upon relapse in many cases, one example being atypical teratoid/rhabdoid tumors, as recently described by Johann, Altendorf et al. [17]. To understand possible prognostic implications of cell density in primary PF-EPN-A, we quantified the proportion of cell-dense areas in nine primary ependymomas and their corresponding relapses, which revealed a mean increase of cell-density in progressive disease (> fourfold increase of the proportion of cell-dense areas in the respective primary). In 8/9 cases, relapses showed a greater proportion of cell-dense areas than the corresponding primaries (Fig. 6a–c). In DNA methylation subtype analysis, 13/32 primary relapse pairings differed in their best-matching subtype. PF-EPN-A1 and 2 subgroup allocation remained constant (Fig. 6d). In *t*-SNE representation with a reference cohort of 337 PF-EPN-A, differences in methylation signature appeared as primaries plotting in a variable distance from their respective relapses. All pairs plotted within the same group of PF-EPN-A1 or 2 reference cases (Fig. 6e). CNV analysis based on DNA methylation data (Fig. 6f) revealed novel chromosome 1q gains and or 6q losses in 16/32 relapses (50%) when compared to the corresponding primary tumors, although such changes did not correlate with the time to recurrence (Suppl. Figure 4, online resource).

## Discussion

Inter- and intratumoral heterogeneity constitutes a research field that has been on the rise in the past years translationally across many tumor entities, delivering new insights in tumor biology and innovative concepts of tumor etiology and progression. Intratumoral heterogeneity is widely recognized as one of the main factors in treatment failure [27], especially in entities with a poor prognosis like glioblastoma [34] and, once understood, holds significant potential for the development of new diagnostic and therapeutic strategies.

PF-EPN-As typically display distinct and often very clearly demarcated high-cell density areas next to areas that consist of more loosely packed tumor cells. Based on this observation, we dissected the intratumoral heterogeneity in a large cohort of PF-EPN-A on an epigenetic, chromosomal, and transcriptomic level and contributed to understanding the different cellularity regions’ biology and clinical implications.

In our histological analysis, we were able to confirm and objectify the impression of high inter- and intratumoral variability in cell density between often neatly demarcated areas in PF-EPN-A. This phenomenon can rarely be found in other pediatric tumor entities of the posterior fossa, especially not in their PF-EPN-B counterparts with otherwise alike histology. However, discrete anaplastic foci of hypercellularity within otherwise low-grade lesions have been described for a number of other CNS tumor entities like oligodendroglioma [1].

Concerning the clinical implications of the spatially differing cellularity, higher proliferative indices in the cell-dense than in the less cell-dense areas suggested a higher biological aggressiveness of these areas. Survival analysis then revealed a significant correlation between the proportion of cell-dense areas of a tumor and decreased overall and progression-free survival, clearly indicating a role in tumor progression and recurrence. Fitting this hypothesis, we also found a higher proportion of cell-dense areas in relapses compared to their primary tumors. The observation of an upward trend in cell density in relapsing tumors is in line with and adds to previous research executed by Yang et al., who described an increasing cell density and proliferation in ependymoma relapses in a relatively small cohort, not differentiating between ependymoma subgroups [47]. Our data confirm this finding in a precise and nuanced measuring approach in a large cohort of PF-EPN-A.

One possible interpretation of the distinct high and low cellularity areas in PF-EPN-A is, that they may represent distinct subpopulations of neoplastic cells. The distinct subpopulations may have arisen throughout tumor progression through the acquisition of additional mutational and epigenetic changes while sharing a common cell of origin. This mechanism has already been suggested for a subset of ependymomas arising from subependymomas of the posterior fossa through subclones with ependymal morphology that eventually supersede the areas of subependymal differentiation [44]. DNA methylation profiles of high and low cell density areas in our cohort were similar, but not identical, suggesting a close relation and therefore suiting the theory of a common cell of origin. However, DNA methylation profiles can also be altered by non-neoplastic cells. Since areas with a low density of tumor cells are likely to have an overall greater fraction of non-neoplastic cells within their samples, it seems important to keep the overall great tumor purity of PF-EPN-A in mind, that applies to high as well as low cell density areas, when interpreting the results. Bulk scRNAseq, H3K27me3-staining and EHZIP-staining have all shown only a very low overall number of other non-neoplastic cells in PF-EPN-A sample that is unlikely to have a significant influence on overall DNA methylation profiles. Consequently, the observed differences in DNA methylation appear hardly explicable solely by different proportions of non-neoplastic cell populations. Thus, we propose that the described differences in DNA-methylation between different cellularity areas are indeed a reflection of epigenetic alterations that accompany the more proliferative cell phenotype in more aggressive areas of the tumor and the distinct transcriptome of these regions.

Indeed, this observation is well in line with a growing body of evidence suggesting that the nine minor subtypes within PF-EPN-A1 and 2 introduced by Pajtler et al. [35] might reflect biological states and plastic changes within ependymomas [9], rather than being well-defined consistent classes. Gillen et al. described PF-EPN-A as usually comprising two or more out of six major scRNA-seq subpopulations, each one with heterogeneous intratumoral localization and a correlation with PF-EPN-A1 and 2 subgroup allocation as well as subtype classification and outcome. They proposed a theory of no-matches on DNA methylation subtype level being mainly the result of this heterogeneity [12]. A similar concept has previously been introduced for glioblastoma. Based on scRNA-seq analyses, four main plastic cellular states of tumor cells in adult and pediatric glioblastoma cases have been described, each resembling a distinct neural cell type. In the respective study, the frequency of states showed inter- and intratumoral heterogeneity, and the preponderance of certain states correlated with the respective subtype determined by the cancer gene atlas (TCGA) [34].

Our results concerning DNA methylation subtype changes between primary tumors and relapses and the observed increase in the prevalence of chromosome 1q gains and 6q losses in PF-EPN-A relapses are consistent with and further support recently published findings by Donson et al. in a partly overlapping cohort. Donson et al. furthermore related the proportion of the subpopulations described by Gillen et al. to ependymoma recurrence and a present chromosome 1q gain and/or 6q loss in the respective samples. In their work, a chromosome 1q gain and/or 6q loss was correlated with a higher abundance of subpopulations of proliferative neuroepithelial progenitors, and a balanced genome with more differentiated subpopulations in their research [9]. Our research complements their findings by being not only based on already relapsed PF-EPN-A but equally including primary tumors without recurrences, as well as by an opposite “top-down” approach from morphology to molecular characteristics. We found an overall higher prevalence of the prognostically unfavourable chromosome 1q gains and 6q losses in the more cell-dense areas. We also observed intratumorally highly variable levels of chromosome 1q gain and 6q loss on a single-cell basis. While we did not explicitly analyze our samples on the proportions of the subpopulations described by Gillen et al., spatial transcriptomic analysis of high and low cell density areas did not show significant differences in the respective marker genes. Nevertheless, given the highlighted interdependencies between Gillen et al.’s, Donson et al.’s and our work, mapping the postulated subpopulations to the different cellularity regions in future work appears to be a promising option to shed further light on the complex biology of PF-EPN-A heterogeneity.

Our data have some important implications for future ependymoma research and diagnostics. To date, histopathology of PF-EPN-A is only taken into account in routine diagnostics to assign WHO grade 2 or 3 with limited prognostic value [25]. The molecular subgroups PF-EPN-A, PF-EPN-B, and PF-SE and further subtypes can only be reliably distinguished by DNA methylation analysis, which today is an essential part of routine diagnostics. So far, methylation analysis is usually performed as a bulk analysis of available tumor material without paying further attention to tumor heterogeneity when sampling.

Our findings strongly suggest histological evaluation of the proportion of cell-dense areas as a potential approach for improving risk stratification in PF-EPN-A. Although it must be borne in mind that assessing the cellularity of the tumors based on a single slide of every available FFPE block may have led to inaccuracies, our results clearly indicate a high proportion of cell-dense areas as a negative prognostic factor in PF-EPN-A. Upon validation of our findings in a larger series, cohorts of patients with PF-EPN-A with more favorable and highly adverse outcomes may be identified better. This might improve the selection of patients who benefit from novel therapy approaches in the future.

Furthermore, our findings indicate that careful sampling of tumor material for subsequent molecular analysis is of utmost importance given the significant differences in DNA methylation, the prevalence of chromosome 1q gain and 6q loss as well as the distinct transcriptome of high and low cell density areas outlined in this research. Judging from our data, the results of a bulk analysis can be assumed to be highly influenced by the areas from which the respective samples are taken. Sampling irrespective of intratumoral heterogeneity may lead to unreliable subtype assignation and possibly to missing important and prognostically relevant information. Although the extent of the effects of different sampling approaches on results and their clinical implications remains subject to future research, routinely taking and analyzing multiple samples from different tumor regions separately instead of bulk analyses seems advisable. Moreover, an approach with a risk stratification based on the most malignant area of a tumor seems reasonable, similar to how it is already done in other tumor entities. Given the higher proliferation and higher prevalence of characteristics that have been shown to be linked to a more unfavorable prognosis, we suggest considering taking samples for diagnostics from the areas with the highest cell density in (suspected) PF-EPN-A [26, 33].

Our research confirms striking inter- and intratumoral heterogeneity within PF-EPN-A and illustrates new insights into significant interdependencies between tumor morphology and clinically relevant molecular hallmarks that should be accounted for when selecting material for routine diagnostics and may prove valuable starting points for future research projects.

### Supplementary Information

Below is the link to the electronic supplementary material.

Supplementary file1 (XLSX 32 kb)

Supplementary file2 (PDF 874 kb)

## Data Availability

All newly generated DNA methylation and Spatial Transcriptomic data presented in this manuscript are available on GEO under the SuperSeries accession GSE248811. Previously published data sets used are referenced with their respective GEO accessions where available.
