# Supplementary material for: Clinically relevant molecular hallmarks of PFA ependymomas display intratumoral heterogeneity and correlate with tumor morphology
Source: Acta Neuropathol. 2024 Jan 24;147(1):23. doi: 10.1007/s00401-023-02682-x (PMC10808473; doi:10.1007/s00401-023-02682-x)
Supplement: Supplementary file 2 — Supplementary file2 (PDF 874 kb) [file 401_2023_2682_MOESM2_ESM.pdf]

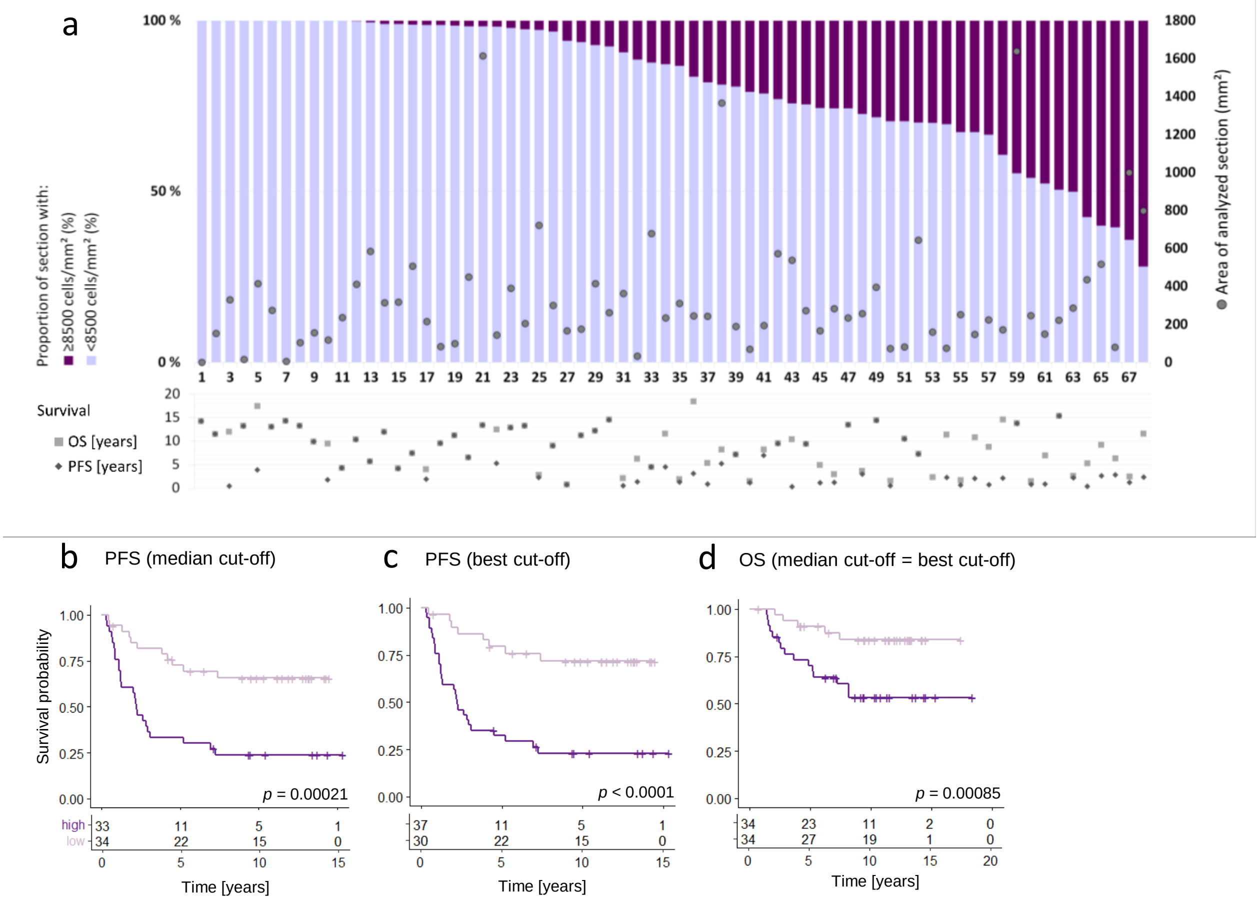

Supplementary Fig. 1

Cellularity and survival in an independant validation cohort of 68 PF-EPN-A.

a Proportion of high and low cell density areas in 68 gross totally resected PF-EPN-A cases from the HIT2000 cohort with the corresponding survival data. **b-d** Kaplan-Meier-Curves of the progression-free and overall survival in 68 tumors (one case with a clear overall survival but unclear relapse status) (b,d) comparing the 34 tumors with a lower proportion of cell-dense areas to the 34 tumors with a higher proportion of cell-dense areas, groups classified as below and above the median, group allocation also represents the cut-off with the highest resulting significance (= smallest *p*-value) for OS, and (c) comparing the 50 tumors with the highest proportion of cell-dense areas to the rest of the cohort, group allocation represents the cut-off with the highest resulting significance (= smallest *p*-value) for PFS.

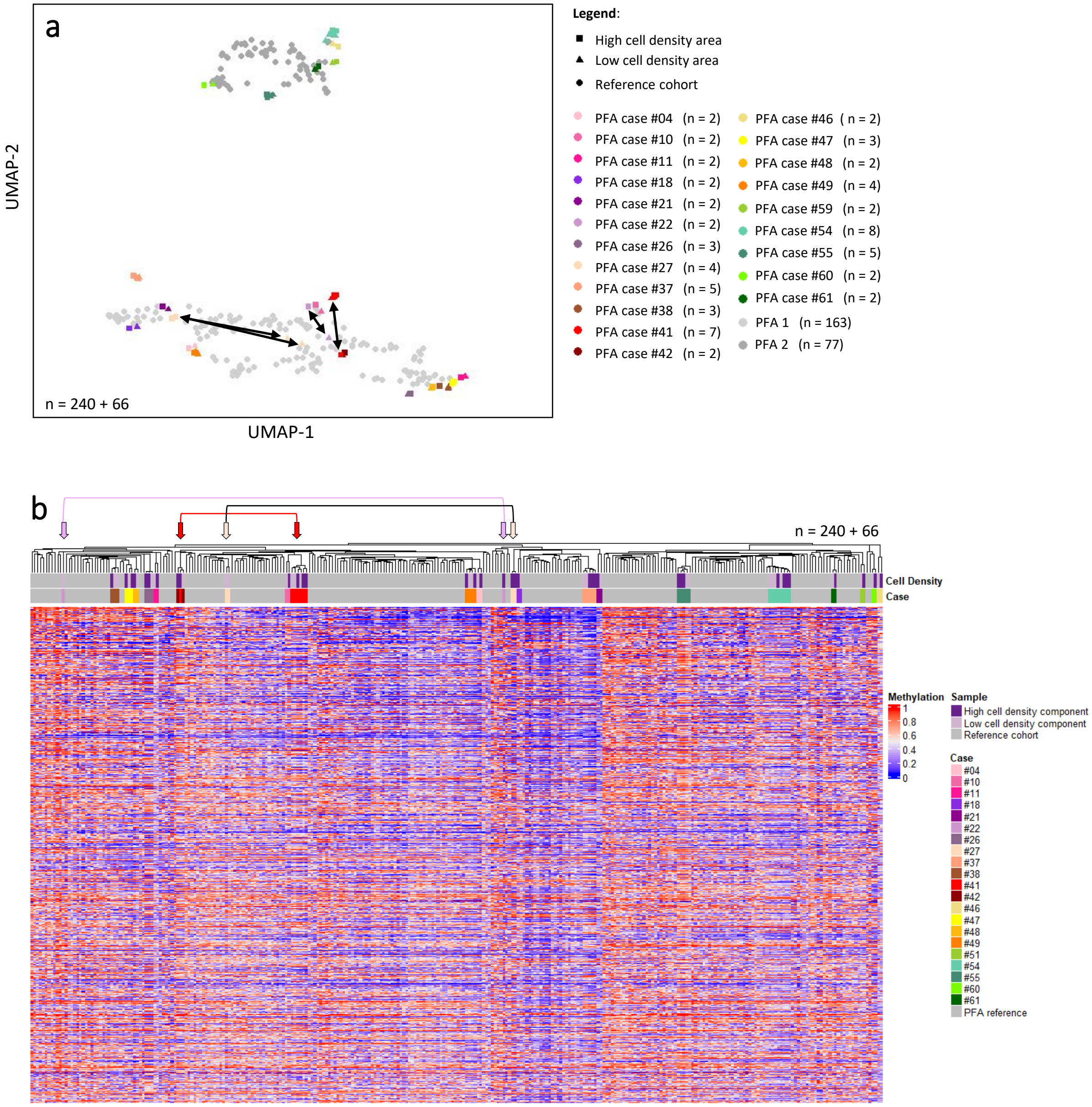

Supplementary Fig. 2

Additional analysis approaches confirm differences in DNA methylation.

a UMAP of low and high cell density area samples of 21 heterogenous PFA ependymomas and PFA ependymomas of the reference cohort as in Figure 2b. **b** Unsupervised clustering heatmap of the same samples as in (a). Cases whose different cell density areas cluster apart from each other are marked with arrows in both panels.

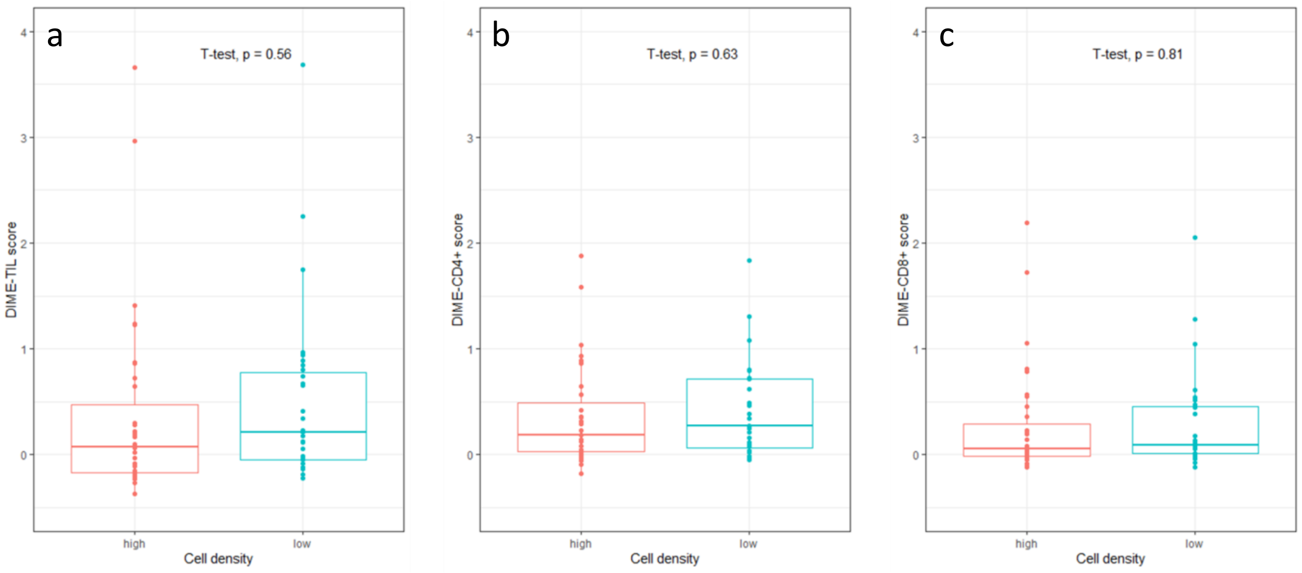

Supplementary Fig. 3

Differential Methylation Analysis for Immune Cell Estimation shows no difference between high and low cell density areas.

a-c Comparison of the estimated immune cell infiltration level between low and high cell density areas of 21 heterogenous PFA ependymomas using (a) DIME-TIL score for tumor-infiltrating lymphocytes, (b) DIME-CD4+ score for CD4+- and (c) DIME-CD8+ score for CD8+ T cell abundance.

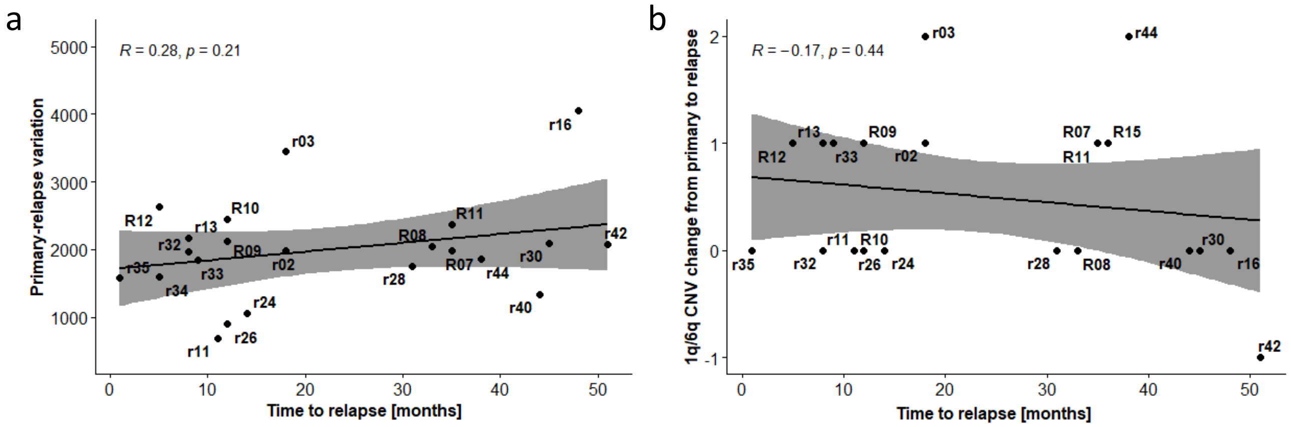

Supplementary Fig. 4

Changes in DNA methylation and CNV status show no significant correlation with time to recurrence.

a Correlation of primary - relapse variance and time to recurrence based on DNA methylation data. Primary - relapse variance was determined by Jensen-Shannon divergence. No correlation was observed on DNA methylation level.

b Correlation of changes in 1q and 6q CNV status from primary to relapse and time to recurrance based on CNV profiles derived from methylation data. No correlation was observed.
